# Responses of Two Scleractinian Corals to Cobalt Pollution and Ocean Acidification

**DOI:** 10.1371/journal.pone.0122898

**Published:** 2015-04-07

**Authors:** Tom Biscéré, Riccardo Rodolfo-Metalpa, Anne Lorrain, Laurent Chauvaud, Julien Thébault, Jacques Clavier, Fanny Houlbrèque

**Affiliations:** 1 Laboratoire d’Excellence « CORAIL», Institut de Recherche pour le Développement, ENTROPIE (UMR9220), BP A5, 98848, Nouméa cedex, New Caledonia; 2 IRD/ R 195 LEMAR, IRD Nouméa, BP A5, 98848, Nouméa cedex, New Caledonia; 3 Université de Brest, Institut Universitaire Européen de la Mer, Laboratoire des sciences de l’environnement marin (UMR6539 CNRS/IRD/UBO), rue Dumont d’Urville, 29280, Plouzané, France; University of Otago, NEW ZEALAND

## Abstract

The effects of ocean acidification alone or in combination with warming on coral metabolism have been extensively investigated, whereas none of these studies consider that most coral reefs near shore are already impacted by other natural anthropogenic inputs such as metal pollution. It is likely that projected ocean acidification levels will aggravate coral reef health. We first investigated how ocean acidification interacts with one near shore locally abundant metal on the physiology of two major reef-building corals: *Stylophora pistillata* and *Acropora muricata*. Two pH levels (pH_T_ 8.02; *p*CO_2_ 366 *μ*atm and pH_T_ 7.75; *p*CO_2_ 1140 *μ*atm) and two cobalt concentrations (natural, 0.03 *μ*g L^-1^ and polluted, 0.2 *μ*g L^-1^) were tested during five weeks in aquaria. We found that, for both species, cobalt input decreased significantly their growth rates by 28% while it stimulated their photosystem II, with higher values of rETR_max_ (relative Electron Transport Rate). Elevated *p*CO_2_ levels acted differently on the coral rETR_max_ values and did not affect their growth rates. No consistent interaction was found between *p*CO_2_ levels and cobalt concentrations. We also measured *in situ* the effect of higher cobalt concentrations (1.06 ± 0.16 *μ*g L^-1^) on *A*. *muricata* using benthic chamber experiments. At this elevated concentration, cobalt decreased simultaneously coral growth and photosynthetic rates, indicating that the toxic threshold for this pollutant has been reached for both host cells and zooxanthellae. Our results from both aquaria and *in situ* experiments, suggest that these coral species are not particularly sensitive to high *p*CO_2_ conditions but they are to ecologically relevant cobalt concentrations. Our study reveals that some reefs may be yet subjected to deleterious pollution levels, and even if no interaction between *p*CO_2_ levels and cobalt concentration has been found, it is likely that coral metabolism will be weakened if they are subjected to additional threats such as temperature increase, other heavy metals, and eutrophication.

## Introduction

Recent studies have highlighted how coral reefs are extremely delicate systems and how they may easily be altered by human activities (e.g. [1]). Among the main sources of pollution threatening them, metals are conservative in nature, persisting in the environment for long periods [2]. Major inputs of heavy metals to the marine environment include urban storm water run-off, industrial effluents, mining operations, and atmospheric contaminants in both particulate and dissolved forms [3]. Furthermore, motor vehicle emissions, disposal of sewage sludge, dredged spoil, ash and antifouling paints for marine vessels and structures also yield appreciable concentrations of heavy metals to the ocean [2,4,5]. Metal pollution affects many reefs worldwide (*e*.*g*. Costa Rica, Panama, Red Sea, Thailand, Tuvalu, Puerto Rico) [5–9]. Among them, coral reefs of New Caledonia (Pacific Ocean) are particularly concerned by this issue. New Caledonia is among the five major nickel producers in the world and extended portions of its fringing reefs are impacted by extensive nickel mining activities [10], which contribute primarily to metal discharges [11–13]. Among these metals, cobalt is associated with nickel in the laterites of the mining sites and the most recent mines in New Caledonia, and throughout the world, launch the mining of cobalt, as a by-product of the treatment of nickel. Experimental studies on metal potential effects on corals mainly investigated their reproduction and early life stages. They can be summarized in the following: inhibition of coral fertilization and reduced reproductive success [14–16], decreased settlement and survival of coral larvae [17]; changes in the rates of photosynthesis resulted in a decrease in coral calcification and growth rates during the juvenile polyp stage [18,19]; loss of zooxanthellae in coral tissue (also called “coral bleaching”) [18,20]; enhanced coral mortality [20,21]. All these experimental studies, using very high metal levels, highlighted the harmful role played by metals when in excess. However, it has never pointed out potential benefic effects due to metals like cobalt (Co), copper (Cu), zinc (Zn), manganese (Mn) and iron (Fe) at moderate and representative levels. These metals are indeed essential for the health and growth of corals since they are cofactors of many enzymes [22,23]. Amongst metals, cobalt might play an important role. For example, the vitamin B_12_, also called cobalamine (due to its cobalt nucleus), is involved in the metabolism of all animals and many phytoplanktonic species [24], and stimulates *in hospite* zooxanthellae growth [25]. Carbonic anhydrases are ubiquitous enzymes known to act as catalysts for the interconversion between CO_2_ and HCO_3_^-^, and it can play an important role when calcification is carbon limited such as under ocean acidification scenarios. However, so far no study has been performed on the direct effect of a cobalt input on coral metabolism.

In addition to waterborne pollutants, the ultimate danger that corals have to face is climate change (e.g. [26]). Atmospheric CO_2_ levels have nearly doubled since pre-industrial times. As CO_2_ diffuses in seawater, it behaves like a weak acid leading to a drop in pH on a global scale [27]. About a third of anthropogenic CO_2_ emissions has been absorbed by the oceans, driving the process of ocean acidification during which absorbed CO_2_ transforms into carbonic acid, increasing the concentrations of H^+^, bicarbonate (HCO_3_^-^), and dissolved carbon dioxide (CO_2_), while lowering carbonate (CO_3_^2-^) concentrations and seawater pH [27,28]. The current consensus estimate is that by the end of this century the rate of calcification in scleractinian corals will decrease by 17–37% as a result of reduced seawater [CO_3_^2-^] due to a doubling of preindustrial levels of atmospheric *p*CO_2_ [29,30] but coral response to acidification is in fact not unequivocal and still puzzling. It has been shown that some tropical species are able to calcify under high *p*CO_2_ levels [31–34]. Comeau *et al*. [35], testing eight tropical species demonstrated that response of tropical coral reef communities to ocean acidification was heterogeneous.

To date, no work has studied the combined effect of ocean acidification and metal impacts on corals. Houlbrèque et al. [33] pointed out that zinc uptake rates in *Stylophora pistillata* were higher under normal pH conditions (pH_T_ 8.1) compared to lower ones (pH_T_ 7.5), suggesting that ocean acidification might also change the incorporation of metals. Depending on whether metals are essential or not, changes in their uptake rates might have radical consequences on coral metabolism.

Here we tested in laboratory conditions the combined effect of ocean acidification and cobalt concentrations on two coral species separately. We also investigated *in situ* the effects of higher cobalt concentrations on coral metabolism through benthic chamber experiments.

## Materials and Methods

### 1-Aquaria experiment

#### Coral collection and experimental setup

*Stylophora pistillata* and *Acropora muricata* were collected in the lagoon of New Caledonia, on the reef of Îlot Maître (22°19.702ʹ S; 166°24.626ʹ E). A license has issued by the “Province Sud” to allow this collection. One hundred twenty terminal portions of branches (2-cm long) for each coral species were cut from 10 parent colonies. After collection, *S*. *pistillata* microcolonies have been hung on nylon wires and suspended on the aquaria, while *A*. *muricata* microcolonies have been glued (Holdfast epoxy) to 2 x 2 cm plastic plates and set up at the bottom of the aquaria. All microcolonies were positioned with the apex in front of the artificial light source. They recovered for one month in the laboratory of the Aquarium des Lagons (Nouméa) under controlled conditions as described below. Microcolonies were randomly assigned to one of the 8 experimental tanks of 20 L volume (*n* = 15 per species per tank) supplied with 100 μm-filtered seawater pumped from 5 meter depth in front of the Aquarium. In each tank, seawater was renewed at a rate of 16.5 L h^-1^ and mixed using a submersible pump (Aquarium system, micro-jet MC 320, Mentor, OH, USA). Temperature (26 ± 0.1°C) and salinity (35.70 ± 0.02) were kept constant using heaters connected to electronic controllers (± 0.2°C accuracy) and routinely verified using an YSI MPS 556 probe (YSI, USA). Corals received a constant irradiance of 120 ± 10 *μ*mol photons m^-2^ s^-1^ (photoperiod was 12h:12h light:dark) using four neon Aquablue plus (Blue-white, 15000 Kelvin, Giesemann, Germany). They were fed twice a week during the recovery period and once a week during the experiment with nauplii of *Artemia salina* (ca. 120 ± 50 *nauplii* L^-1^).

After the recovery, four tanks were set-up at ambient pH (pH_T_ 8.02 ± 0.03; *p*CO_2_ 366 μatm) and four others at pH level projected for the end of the century (pH_T_ 7.75 ± 0.06; *p*CO_2_ 1140 *μ*atm) (IPCC 2007). pH was controlled using a pH-stat system (IKS, Karlsbad, accuracy ± 0.05 pH unit) by bubbling independently pure CO_2_ in each tank that were continuously aerated with CO_2_-free air. For each *p*CO_2_ condition, two tanks received a natural cobalt concentration of the seawater pumped in front of the Aquarium (Natural: 0.035 ± 0.007 *μ*g L^-1^), and two others were enriched in cobalt (Polluted: 0.222 ± 0.006 *μ*g L^-1^). For that, a peristaltic pump (ISMATEC), together with seawater flow-through, continuously supplied the experimental tanks with solution of stable cobalt (CoNO_3_, CPAchem, Bulgaria) at a rate 50 ml h^-1^. Throughout the experiment, cobalt concentrations have been analyzed once a week by the AEL laboratory (*Analytical Environmental Laboratory*) of Nouméa, according to the protocol described in [36].

Colonies were maintained under these experimental conditions for five weeks, after which their photosynthetic efficiency and growth rates were measured.

#### Seawater carbonate chemistry

Seawater pH values were continuously monitored by a pH-stat system, which electrodes were calibrated using NBS solutions (Seawater National Bureau of Standards) and adjusted every day to the desired pH_T_ (Total Scale) using a pH meter with a glass electrode (Eutech Instruments EcoScan) calibrated with Tris/HCl referenced solutions ([37]; standards provided by A.G. Dickson, batch 13). Mean pH_T_ were calculated from hydrogen ion concentrations of each measurement and then re-converted back to pH [37]. Total alkalinity was measured twice a week on water samples collected in glass bottles, filtered at 0.45 μm (GF/F Whatman) and stored in the dark at 4°C to avoid biological alteration. The pH was measured at 0.1 ml increments of 0.01 N HCl at 25°C using a Metrohm titration system (848 Titrino Plus). Three replicated 20 mL sub-samples were analyzed. Total alkalinity (A_T_) was calculated from the Gran function applied to pH variations from 4.2 to 3.0 as mEq L^-1^ from the slope of the curve HCl volume versus pH. Titrations of A_T_ standards provided by A.G. Dickson (batch 121) were within 0.85 *μ*mol kg^-1^ of the nominal value. Mean A_T_ of seawater was 2.341 ± 0.047 mmol kg^-1^ (*n* = 40). CO_2_ and saturation states of aragonite (Ω_ara_) were calculated from pH_T_, mean A_T_, temperature, and salinity using the free access CO_2_ Systat package.

#### Photosynthetic efficiency measurements

Photosynthetic efficiency (F_v_/F_m_) and the relative electron transport rate (rETR) of the Photosystem II (PSII) of zooxanthellae *in hospite* were measured using a DIVING-PAM fluorometer (Walz, Germany) (*n* = 10 for each species and each tank). During measurements light sources were switched off. Ambient light level at the aquarium room was<10 *μ*mol photon m^-2^ s^-1^ and did not substantially affect the coral F_v_/F_m_. After 15 min of dark adaptation (after [38]), the first measurements were performed. The initial fluorescence (F_0_) was measured by applying a weak pulsed red light (3 *μ*s, LED 650 nm) on dark-adapted colonies. A saturating pulse (800 ms) of bright actinic light (8,000 *μ*mol photons m^-2^ s^-1^) was then applied to give the maximum fluorescence value (F_m_). Variable fluorescence (F_v_) was calculated as F_m_-F_0_. At the end of all F_v_/F_m_ measurements, light was switched on and the corals were allowed to recover under light conditions for 1 h after which rapid light curves (RLC) were generated by illuminating corals for 10·s periods, eight times from 0 to ca. 3000 *μ* mol·photon·m^-2^·s^-1^, and the coral maximum relative electron transport rate (rETR_max_) was assessed and compared between the different treatments.

During measurements, the 8·mm optical fibre was maintained perpendicular to the coral’s surface using a black-jacket at a fixed distance of 5·mm [39]. This system created a quasi-darkness status allowing the rapid re-oxidation of primary electron acceptor [40]. The same setting was also used during F_v_/F_m_ measurements to guarantee correct distance of the optical fiber to the coral.

It is worth noting that to calculate ETR using RLCs, some assumptions were made. First, because we did not measure the different tissue absorptances between species, we used the relative ETR (*sensu* [40]) which does not account for the fraction of incident light absorbed [41]. rETR was therefore uniquely used to compare data from different treatments and not between species. Another source of significant errors when measuring rETR is a likely different symbiont density between samples which can lead to a significant change in reflected light and thus in absorptance. As we measured ETR only one time at the end of the experiment and as the zooxanthellae density did not change between treatments (see Table 1), we are confident that our measurements of rETR reflect coral response to treatment uniquely. It should be noted that pre-configured protocols designed for measuring ETR by PAM fluorometry permit rapid assessment of the light adapted state of corals without allowing sufficient time for steady state conditions to be established, which is required to correctly evaluate the photosynthetic response to light (i.e. ETR vs Irradiance curve [42]). However, our approach was to contrast ETRs between different treatments and therefore contrast relative differences in the responses observed. In no way we measured absolute electron transport rates to contrast relative patterns in light utilization of the two studied species.

**Table 1:** Summary of two-way ANOVAs testing, in aquaria, the effect of *p*CO_2_ (366 and 1140 ppm) and cobalt concentrations (Natural, 0.03 *μ*g L^-1^ and Polluted, 0.2 *μ*g L^-1^) on *Stylophora pistillata* and *Acropora muricata* main physiological parameters.

	*Stylophora pistillata*				*Acropora muricata*			
Source of variation	SS	df	F-ratio	*p*-Values	SS	df	F-ratio	*p*-Values
**Zooxanthellae**								
*p*CO_2_	948x10^8^	1	1.92	0.18	820x10^8^	1	2.23	0.15
Cobalt	320x10^8^	1	0.65	0.43	531x10^8^	1	0.014	0.91
*p*CO_2_ x cobalt	21x10^10^	1	4.33	0.51	136x10^8^	1	0.37	0.55
Error	987x10^9^	20			736x10^9^	20		
**Chlorophyll *a***								
*p*CO_2_	12.96	1	6.54	0.019*	2.969	1	1.837	0.19
Cobalt	6.74	1	3.40	0.08	0.627	1	0.388	0.54
*p*CO_2_ x cobalt	24.55	1	12.38	0.002*	0.002	1	0.001	0.927
Error	39.64	20			32.31	20		
**ETR**_**max**_								
*p*CO_2_	1569	1	13.15	0.001*	253	1	4.14	0.049*
Cobalt	1950	1	16.35	0.000*	1000	1	16.38	0.000*
*p*CO_2_ x cobalt	633	1	5.30	0.027*	8	1	0.13	0.721
Error	4295	36			2198	36		
**F**_**v**_ **/F**_**m**_								
*p*CO_2_	0.002	1	1.555	0.22	0.002	1	4.040	0.052
Cobalt	0.006	1	6.27	0.017*	0.001	1	1.858	0.181
*p*CO_2_ x cobalt	0.000	1	0.012	0.912	0.000	1	0.956	0.335
Error	0.035	36			0.015	36		
**Growth rate**								
*p*CO_2_	0.01	1	0.00	0.956	0.08	1	0.038	0.845
Cobalt	68.37	1	28.71	0.000*	16.45	1	8.210	0.006*
*p*CO_2_ x cobalt	5.48	1	2.30	0.135	1.11	1	0.553	0.46
Error	123.83	52			104.19	52		

* face numbers indicate *p*<0.05.

#### Growth rates

Microcolonies were weighted (*n* = 7 for each species and each tank) using the buoyant weight technique [43]. Samples were weighed using a Mettler AT200 electronic balance (readability 0.1 mg) in seawater of known density as measured by an YSI MPS 556 probe (YSI, USA). For *A*. *muricata*, for which samples were attached to plastic plates, they were weighed before and after attachment to tagged plastic plates, and the difference (plate and glue weight) was subtracted from the total weight. The net buoyant weight of the corals (total coral weight *minus* the weight of each plate) was converted into dry weight using the density of the pure aragonite (2.94 g cm^-3^). Their net calcification rates were calculated as the daily change in dry weight between the initial and the final weight and expressed in mg g^-1^ d^-1^.

#### Zooxanthellae and chlorophyll content

At the end of the incubation microcolonies (n = 3 per each species and tank) were collected and frozen (-20°C) for zooxanthellae and total chlorophyll (chl) measurements. Tissue was removed from coral colonies using an air pick [44] and the slurry was homogenised with a Potter tissue grinder. For each sample, the number of zooxanthellae was counted five times by light microscopy using a Neubauer’s cell. Chlorophylls were extracted twice in 100% acetone (24 h at 4°C). The extracts were centrifuged at 10,000 g for 15 min and the absorbances were read at 630, 663, 750 nm. Chlorophyll concentrations were computed according to the spectrometric equations of [45]. All measurements were normalized to the nubbin surface area, which was measured using the aluminum foil technique [46].

### 2—*In situ* experiment

**Fig 1:**
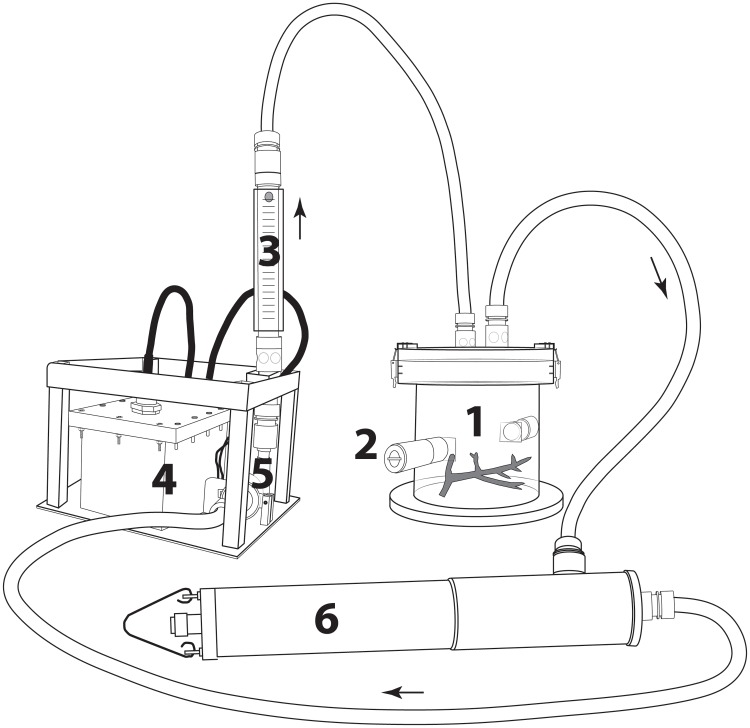
Schematic representation of the experimental system. 1- Benthic chamber with a coral colony, 2- Seawater sampling port, 3- Flow meter, 4- Battery in a waterproof housing, 5- Adjustable submersible pump, 6- Multiparameter probe. The arrows indicate the direction of the water flux.

*In situ* experiments were conducted during two consecutive sunny days at the end of August 2012 during the winter Austral season. The study site was located at Tabou Reef (22°28.845ʹ S; 166°26.806ʹ E) in the southwest New Caledonia lagoon at a depth of 5 m. Three colonies of ca. 15 cm long of *A*. *muricata* were sampled by scuba diving near to the study site. Their bare skeleton (due to the collect) was covered by a layer of Holdfast epoxy to avoid the development of algae on this part. Specimens were then transferred to benthic chambers left opened until the beginning of the experiment. Four transparent PVC benthic chambers of 0.19 m diameter and 6.4 L volume were used simultaneously, three chambers containing *A*. *muricata* colonies and a control one without coral colony to account for seawater microbial activity. Each chamber was hermetically connected to an YSI 6920 multiparameter probe. Seawater was recirculated between the chamber and the probe at a water flow of 2 L min^-1^ using an adjustable submersible pump alimented by waterproof batteries [47] (Fig 1). Photosynthetically active radiation (PAR, 400–700 nm) irradiance (I, *μ*mol photons m^-2^ s^-1^) was measured adjacent to experimental chambers using quantum sensors (LI-192 SA coupled to a Li-1400 LI-COR). Oxygen, temperature, salinity and depth were recorded every minute inside each chamber with the YSI 6920 multiparameter probe.

During two consecutive days, for each species, six successive incubations of one hour were performed from 11:00 am to 9:00 pm, to encompass the full range of daily irradiance levels, including dark. The first series of incubations has been realized at seawater natural cobalt concentration (less than quantification limit measured by [36]). The second series of incubations have been performed on the same coral colonies but 10 ml of a 17 *μ*mol l^-1^ cobalt solution have been injected in each chamber at the beginning of each incubation (CoNO_3_, CPAchem, Bulgaria). Final cobalt concentration into the chambers was equal to 1.06 ± 0.16 *μ*g l^-1^ (i.e. Polluted). Between incubations, enclosures were left open for at least 30 mn to restore ambient conditions.

#### Photosynthetic, respiration and calcification rates

Photosynthetic and respiration rates, as well as calcification rates associated to *A*. *muricata* metabolism were measured during the in situ incubations at different light levels and in the dark. At the beginning and at the end of each of the six incubations, seawater samples were collected with 450 ml syringes in each chamber for pH and total alkalinity (A_T_) measurements. pH was measured immediately on board using a pH-meter (Radiometer pH240) calibrated with TRIS/HCl (2-amino-2-hydroxymethyl-1, 3-propanediol) and 2-aminopyridine/HCl buffer solutions in synthetic seawater of salinity 35. Samples were then filtered through 0.7 *μ*m Whatman glass-fiber filters (GF/F) and stored in 250 ml bottles in the dark. Total alkalinity (*μ*mol kg^-1^) was further determined on 20 ml subsamples (6 replicates) by Gran automatic potentiometric titration (Radiometer, Titrilab TIM 865) using 0.01 M HCl (see above “Seawater carbonate chemistry” for further details). Total alkalinity values were corrected for ammonium concentrations which were analyzed on board with the fluorometric method [48] (Turner Trilogy fluorometer). These concentrations were negligible and did not modify A_T_ values. The mean standard deviation of replicate measurements was less than 0.003 mmol l^-1^.

Calcification rates at each light level and in the dark were estimated using the alkalinity anomaly technique [49] as the difference between the final and the initial A_T_ values of each incubation. Calcification rates (Cnet, *μ*mol CaCO_3_ h^-1^) were estimated for each incubation using the following equation:

$$C_{net}=\frac{\Delta A_{T}\times v}{\Delta t_{}\times 2}$$ (1)

where ΔA_T_ is the variation of total alkalinity during incubation (*μ*mol L^-1^), v is the volume of the benthic chamber (L) and Δt is the incubation time (h).

Oxygen production and consumption rates were measured by the oxygen sensor of the YSI probe which was calibrated before each experiment against air saturated with moisture. Rates of photosynthesis and respiration were estimated by regressing oxygen data against time. Net photosynthetic rates were calculated according to the following equation:

$$P_{net}\;=\;(\Delta {\text{O}}_{2}\;\times \text{ v})$$ (2)

where P_net_ is the rate of net photosynthesis (*μ*mol O_2_ h^-1^); v is the volume of the chamber (L); Δ[O_2_] is the dissolved oxygen variation (*μ*mol O_2_ L^-1^ h^-1^) during the 1 h incubation.

At the end of all the incubations coral colonies were frozen (-20°C) for further analyses. Photosynthesis, respiration and calcification rates were normalized by unit surface skeleton (cm^-2^), which was measured using measured using the aluminum foil technique [46].

For each colony, a Michaelis-Menten model [50,51] was iteratively fitted using non-linear regression (Gauss-Newton algorithm) to P_net_ versus *in situ* PAR irradiance (I, *μ*mol photons m^-2^ s^-1^), according to the following equation:

$$P_{net}=\frac{P_{max}\times I}{K_{m}+I}-R$$ (3)

where P_max_ is the gross maximal production rate (*μ*mol O_2_ cm^-2^ h^-1^), K_m_ is the half saturation constant (*μ*mol photons m^-2^ s^-1^), and R is the respiration in the dark (*μ*mol O_2_ cm^-2^ h^-1^).

From the equation above, P_max_ and K_m_ have been calculated (Eqs 4 and 5):

$$P_{\max}=\frac{\left(P_{net}\;+\;R)\;\times \;(K_{m}\;+I\right)}{I}-R$$ (4)

$$K_{m}=\frac{\left(P_{max}\times I\right)}{C_{net}+R}-I$$ (5)

The same procedure was also used to fit calcification rates (C_net_) versus Irradiance values. Eqs 6 and 7 were therefore used to calculate maximum fitted calcification values (C_max_) as follow:

$$C_{net}=\frac{C_{max}\times I}{K_{m}+I}-C_{dark}$$ (6)

$$C_{\max}=\frac{\left(C_{net}\;+\;C_{dark})\;\times \;(K_{m}\;+I\right)}{I}-C_{dark}$$ (7)

where C_max_ is the gross maximal calcification rate (*μ*mol CaCO_3_ cm^-2^ h^-1^), K_m_ is the half saturation constant (*μ*mol photons m^-2^ s^-1^), and C_dark_ is the calcification rate measured in the dark (i.e. dissolution, *μ*mol CaCO_3_ cm^-2^ h^-1^).

### Statistical analysis

For the aquaria experiment, three-way ANOVA factorial analyses were used to test the effects of pH (normal and low), cobalt (normal and polluted) and tanks (two replicates), independently for each of the two species. All data were tested for the assumptions of normality and homoscedasticity using Shapiro Wilk's test and Cochran's c-test respectively. After verification of the absence of significant differences between tanks (ANOVA, p > 0.05), data were pooled before proceeding to test for main effect using two-way ANOVAs. Two-way ANOVAs were used to test the effects of pH_T_ and cobalt concentrations on zooxanthellae density, chl concentration, net calcification rate, photosynthetic efficiency (F_v_/F_m_) and maximum electron transport rate (rETR_max_). All the tests were performed using PRISM software (Statsoft). When the ANOVA determined a significant difference, a Tukey’s honest significant difference test (HSD) was used to attribute differences between specific factors. All data are expressed as the mean ± SD. For each *in situ* experiment P_max_, K_m_ and R, as well as C_max_ and C_dark_ were compared between cobalt concentrations (i.e. natural vs. polluted) using F-test according to [52].

## Results

### 1-Aquaria experiment

Zooxanthellae density did not differ between experimental treatments (2-way ANOVA, p > 0.05; Table 1) for both *S*. *pistillata* (pooled data, 1.06 x 10^6^ ± 2.26 x 10^5^ cells cm^-2^) and *A*. *muricata* (pooled data, 1.21 x 10^6^ ± 1.84 x 10^5^ cells cm^-2^). Chl *a* concentrations slightly but significantly varied between *p*CO_2_ (6.04 ± 1.9 and 7.10 ± 1.1 *μ*g chl cm^-2^ at pH_T_ 8.1 and 7.8 respectively) and its interaction with cobalt (6.04 ± 1.9 and 7.10 ± 1.1 *μ*g chl cm^-2^ at Natural and Polluted concentrations respectively) for *S*. *pistillata* (interaction *p*CO_2_ x Metal F_1,20_ = 12.38, p = 0.002; Table 1), but not for *A*. *muricata* (pooled data: 6.389 ± 1.242 *μ*g chl cm^-2^; Table 1).

**Fig 2:**
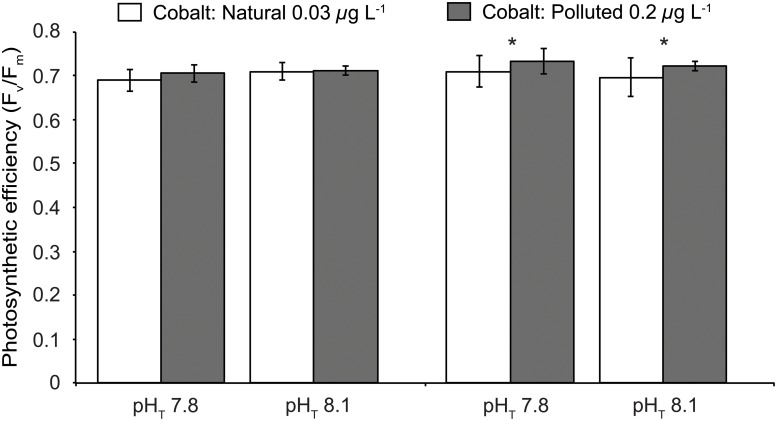
Effective photosynthetic efficiency (F_v_/F_m_) measured on dark adapted *Stylophora pistillata* and *Acropora muricata* colonies exposed during five weeks to two pH_T_ conditions (pH_T_ 7.8 or 8.1) and two cobalt concentrations (Natural and Polluted). Stars indicate statistically significant differences (Tukey’s test, *p*<0.05). Each point represents the mean ± SD (*n* = 10).

**Fig 3:**
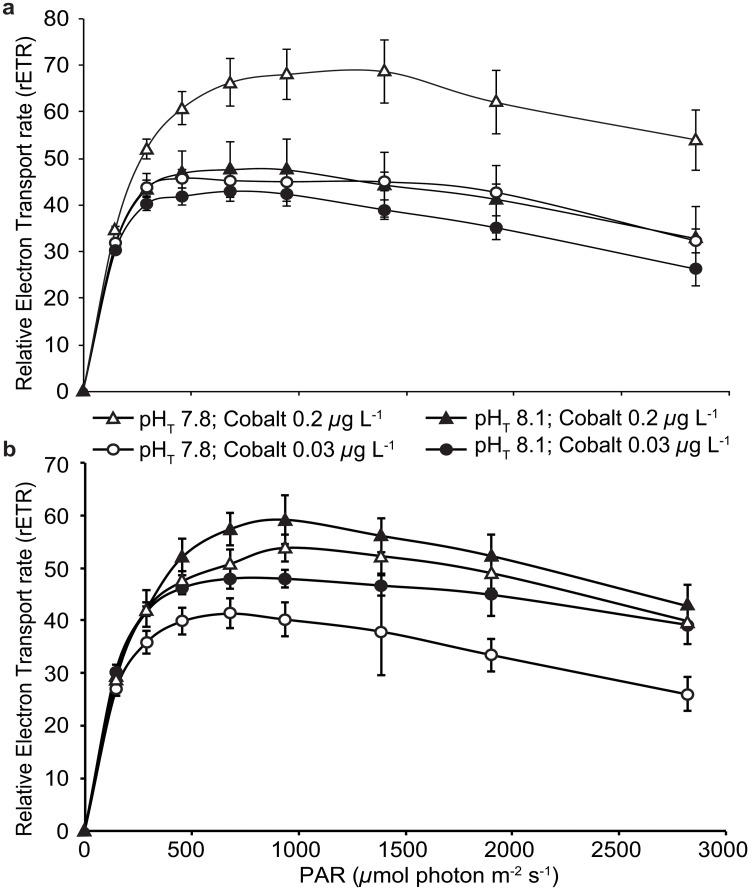
Relative electron transport rate (rETR) versus irradiance (PAR) for colonies of *Stylophora pistillata* (a) and *Acropora muricata* (b) (mean ± SD, *n* = 10) exposed during five weeks to two pH_T_ conditions (pH_T_ 7.8 or 8.1) and two cobalt concentrations (Natural and Polluted).

Concerning their photosynthetic properties, both coral species did not show uniform responses to experimental treatments (Figs 2 and 3). For *S*. *pistillata*, F_v_/F_m_ and rETR_max_ significantly differed between treatments (2-way ANOVA, p<0.05, p<0.01 respectively; Table 1). F_v_/F_m_ was not affected by *p*CO_2_ treatment (2-way ANOVA, p > 0.05; Table 1) but was enhanced by cobalt with higher values at the polluted metal concentration (Tukey test, p<0.05), while rETR_max_ values showed an interaction between *p*CO_2_ and cobalt (2-way ANOVA, F_1,36_ = 5.30, p = 0.027; Table 1). On the opposite, *A*. *muricata* F_v_/F_m_ were not affected by both cobalt concentration and *p*CO_2_ treatments (2-way ANOVA, p > 0.05; Table 1), while rETR_max_ values for this species significantly varied between treatments (2-way ANOVA, p > 0.05; Table 1). They increased at polluted cobalt concentrations but decreased at elevated *p*CO_2_ level (Tukey test, p<0.01 and p<0.05 respectively) (Fig 3).

**Fig 4:**
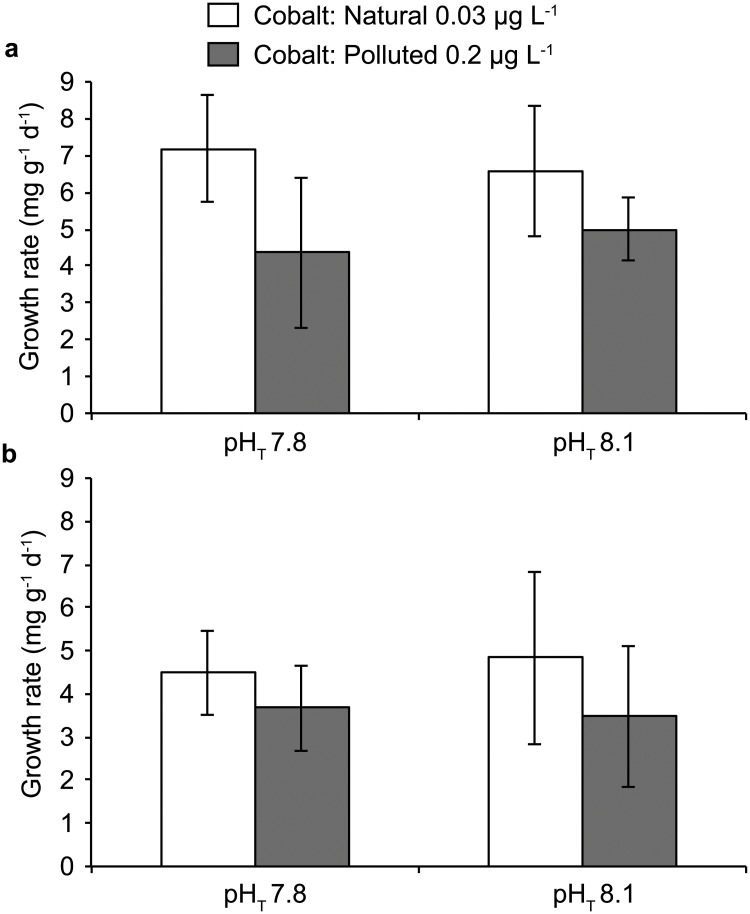
Growth rate (mg g^-1^ d^-1^) measured for *Stylophora pistillata* (a) and *Acropora muricata* (b) colonies after five weeks of incubation under two pH_T_ conditions (pH_T_ 7.8 or 8.1) and two cobalt concentrations (Natural and Polluted). Data are mean ± SD (*n* = 14).

Coral growth rates for both species were not significantly affected by *p*CO_2_ treatments but significantly by cobalt (2-way ANOVA, p > 0.05; Table 1 and Fig 4). Polluted cobalt concentrations significantly decreased their growth rates (Tukey test, p<0.01) with values of 4.68 ± 1.44 mg g^-1^ d^-1^ and 6.88 ± 1.62 mg g^-1^ d^-1^ for *S*. *pistillata* and 3.57 ± 1.31 and 4.66 ± 1.48 mg g^-1^ d^-1^ for *A*. *muricata* at polluted and natural cobalt concentration respectively.

### 2—*In situ* experiment

**Fig 5:**
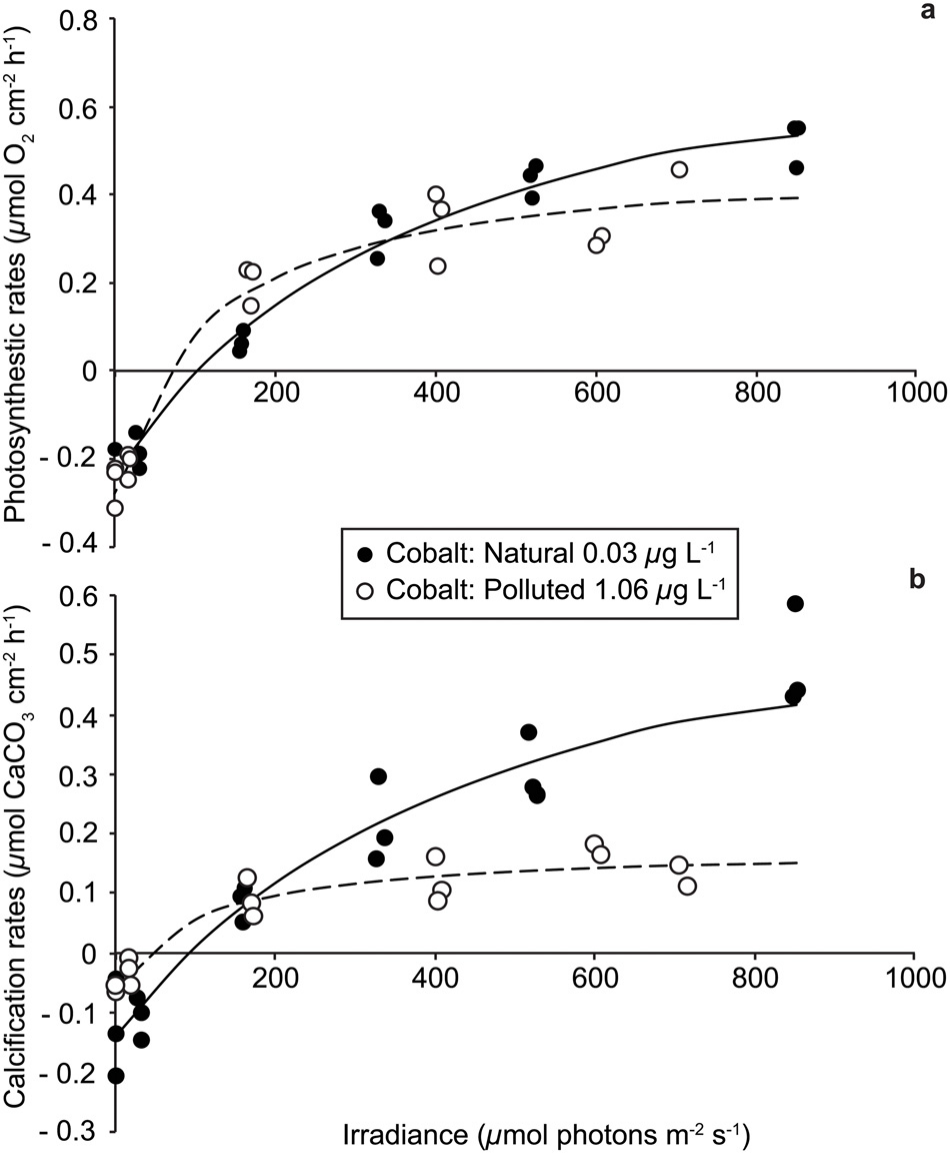
*Acropora muricata* photosynthetic (a) and calcification rates (b) measured *in situ* during benthic chamber incubations at Natural (<0.03 *μ*g L^-1^) and Polluted (1.06 *μ*g L^-1^) cobalt concentrations.

Rates of maximal gross photosynthesis (P_max_) per unit surface area were significantly lower on *A*. *muricata* incubated with cobalt than exposed to natural concentrations (0.77 vs. 1.16 μmol O_2_ cm^-2^ h^-1^; F-test = 15.05, p = 0.000; Fig 5a). Cobalt also significantly affected (F-test = 12.51, p = 0.001) the half saturation constant (K_m_), with much lower values measured for colonies incubated with a cobalt supply (115.29 vs. 408.41 *μ*mol photons m^-2^ s^-1^). No significant differences in the respiration rates were found (R = -0.28 vs. -0.24 μmol O_2_ cm^-2^ s^-1^, respectively) (F-test = 15.05, p > 0.05).

Cobalt significantly affected the growth of *A*. *muricata* (Fig 5b), with both maximal light calcification (C_max_: F-test = 6.33, p = 0.02) and dark calcification rates (C_dark_: F-test = 4.80, p = 0.04) lower for corals submitted to cobalt (0.120 and 0.030 μmol CaCO_3_ cm^-2^ h^-1^ for C_max_ and C_dark_ respectively) than in natural seawater (0.453 and 0.070 μmol CaCO_3_ cm^-2^ h^-1^ for C_max_ and C_dark_ respectively).

## Discussion

Ocean acidification is one of the major threats that tropical coral reefs are facing during this century. Key life functions in corals, such as growth and photosynthesis, have been shown to be affected by high *p*CO_2_ levels (i.e. [27,30]). In addition, near shore coral reefs are often already weakened by coastal human impacts, such as metal pollution, but the combination of ocean acidification and these other anthropogenic pollution forms has not been investigated yet. Thus, this study *(i)* examined, in laboratory conditions, the responses of two corals to the combination of metal pollution (here the cobalt) and ocean acidification and *(ii)* determined *in situ* whether a cobalt input alters the main coral physiological parameters. Cobalt levels added into the experimental tanks and the benthic chambers are realistic since they are in the range of maximal concentrations measured along the coasts of New Caledonia [36]. Our experiments highlighted that even a moderate cobalt concentration (0.2 *μ*g L^-1^), and without interaction with acidification, adversely affects the growth of both coral species and that an increase in cobalt concentration to 1.06 *μ*g L^-1^ leads not only to coral growth decrease, but also to an inhibition of their photosynthesis process.

### Effect of cobalt enrichment on coral physiology

One of the major results of this study is that, at cobalt concentrations regularly measured along the New Caledonian coasts for example (0.2 *μ*g L^-1^) [36] a decrease of 28% in growth rates is already observed for both species. Furthermore, *in situ* incubations of *A*. *muricata* colonies at a higher concentration (ca. 1 *μ*g L^-1^) confirmed these results, revealing coral calcification rates of 70% lower than at normal cobalt concentrations. Although no study so far have measured the effects of cobalt on coral growth, our result is in agreement with [53] which demonstrated, for a coccolithophoridae species, *Cricosphaera carterae* that a cobalt concentration of 200 *μ*moles L^-1^ leaded to a decrease in calcium ion incorporation rates. Therefore, our results suggest that near shore reef corals may greatly suffer from metal pollution and, in turn, may be less prone to resist to other anthropogenic stresses.

As calcification and photosynthesis mechanisms are coupled in symbiotic corals [29], one should expect that the decrease in calcification rates observed in the present study was combined to an inhibition of the photosynthetic efficiency and/or the zooxanthellae. However, during the laboratory experiment, the F_v_/F_m_ measured in *S*. *pistillata* and *A*. *muricata* (ranging from 0.6 to 0.7), exposed to an ecologically relevant enrichment in cobalt (0.2 *μ*g L^-1^), revealed that their photosynthetic processes were stimulated by such moderate cobalt input and were operating at their maximum capacity [54]. Rather, this moderate cobalt concentration stimulates the rETR_max_ values in *S*. *pistillata* and *A*. *muricata*. On the other hand, their zooxanthellae concentrations did not change, suggesting that, unlike iron [19], cobalt addition did not stimulate symbiont densities but would rather stimulate the photosynthetic efficiency of these algal cells. This is consistent with previous studies performed on the same coral species, showing a stimulation of rETR_max_ values in response to a moderate zinc or iron input [19,55]. Two hypotheses may explain this stimulation: *(i)* as cobalt is a cofactor of the carbonic anhydrase, an additional input would stimulate this enzyme and would bring additional inorganic carbon to the photosynthetic process [56,57]; *(ii)* as coral gastric activity harbors a bacterial community able to produce the B12 vitamin, named cobalamin (due to its cobalt core), an enhancement of this vitamin production could stimulate the photosynthetic process [58].

When cobalt was equal to ca. 1 *μ*g L^-1^, in the *in situ* experiments, photosynthetic rates per unit surface area decreased, suggesting that the toxic threshold for zooxanthellae in *A*. *muricata* was reached. This result is consistent with previous studies, in which a clear inhibition of the photosynthetic efficiency of the two branching corals *Acropora cervicornis* and *Pocillopora damicornis* was observed, when they are exposed to elevated copper levels between 4 and 20 *μ*g L^-1^ [59]. Furthermore, as metals are mostly accumulated in coral tissues and more particularly in symbiotic algae [55], their expulsion is thought to be a mechanism of metal detoxification. Although our *in situ* incubations were too short to detect a change in the zooxanthellae density and no measurements have been performed, it is likely that the cobalt level tested reached the zooxanthellae toxic threshold affecting whole coral metabolic functions. Zooxanthellae expulsion was observed for *Acropora formosa* and *Porites lutea* exposed to extreme copper and iron concentrations, from 10 to 100 times higher than the concentrations measured in coral reef surrounding waters [18,60]. In conclusion, our results show that while extreme cobalt concentrations may be lethal for corals, reducing calcification and photosynthetic metabolism, a moderate concentration effect may be more species-specific. Two hypotheses may likely explain this antagonism: *(i)* algae and host may be in competition for inorganic carbon for photosynthesis and calcification [19,61,62] but the coral ability to compete for this substrate may be highly species-specific with some species able to preferentially allocate inorganic carbon to the photosynthetic process; *(ii)* moderate cobalt concentration might be toxic for the host cells but not yet for the zooxanthellae. Despite these observations, identifying the mechanisms underlying the enhancement of the photosynthetic process or the calcification decrease is beyond the scope of our study and we suggest that further research should be conducted.

### Effect of pCO_2_ increase on coral physiology

Higher *p*CO_2_ levels did not affect the photosynthetic efficiency of both coral species but caused an opposite effect on their rETR_max_ values, stimulating *S*. *pistillata* and inhibiting *A*. *muricata*. Previous studies focused on the effects of ocean acidification on photosynthetic processes gave ambiguous results. While the lack of impact for *S*. *pistillata* have been already noticed during short-term studies [33,63–66] and are consistent with the assertion that corals do not rely on dissolved CO_2_ for their photosynthesis [29]; other studies rather highlighted a detrimental effect of *p*CO_2_ on the coral photosynthetic efficiency [67,68]. Response heterogeneity among corals would be linked to the symbiont types [69].

No change in the growth rates of *S*. *pistillata* and *A*. *muricata* was detected between the different *p*CO_2_ conditions. These results confirm a recent study performed on *S*. *pistillata*, where all coral fragments submitted for one month to high *p*CO_2_ conditions (2,039 *μ*atm) survived and showed equivalent calcification rates to corals submitted to normal conditions [33]. Although these results should be taken with caution, since corals were incubated during short periods to low pH, it is consistent with some previous studies showing that some corals are able to calcify at low-pH seawater (e.g. [32,44,70–73]). Coral species like *S*. *pistillata* and *A*. *muricata* would be part of a “low-sensitivity” group to *p*CO_2_ [74] and to the “potential winners” that would be able to control pH at their calcification site [73] and would outcompete species unable to do it [34]. Furthermore, ocean warming should also to be taken into consideration as [64] showed that *S*. *pistillata* was insensitive to doubled *p*CO_2_ at 25°C but on the other hand experienced a 50% reduction in calcification rate at a higher temperature (+ 3°C). So, based on changes in temperature, some coral species like *S*. *pistillata* could shift between insensitivity and high sensitivity to *p*CO_2_ increase.

Even if no interaction between *p*CO_2_ levels and cobalt concentration has been revealed in our study for these two coral species, it is likely that coral colonies, for which growth rates already decrease by 28% when submitted to a moderate cobalt input, will be weakened if they are subjected to additional threats (i.e. temperature increase, other metal pollution, eutrophication). Indeed, when their temperature threshold has been exceeded or when corals are submitted to a poor seawater quality, it has been widely demonstrated that they stop their growth and expel their zooxanthellae (e.g. [62,75,76]) and so a cobalt pollution would exacerbate these phenomena.

It is also likely that species originally classified into the “highly sensitive group” such as *Porites lutea* or *Acropora cervicornis* for example [77] will be probably more drastically affected by metal inputs. In addition, our results only correspond to a five weeks incubation experiment and as it has been demonstrated that metals accumulate in coral tissues [55], a longer-term experiment should induce a stronger calcification inhibition and an overrun of the toxicity threshold for the zooxanthellae. Previous studies performed on other marine organisms like copepods and polychaetes demonstrated that metal pollution increases their sensibility to ocean acidification [78,79] and the present study clearly indicates that it is also the case for corals.
